# Coordinated Control of Slip Ratio for Wheeled Mobile Robots Climbing Loose Sloped Terrain

**DOI:** 10.1155/2014/396382

**Published:** 2014-09-03

**Authors:** Zhengcai Li, Yang Wang

**Affiliations:** School of Mechanical Instrumental Engineering, Harbin Institute of Technology, Harbin 150001, China

## Abstract

A challenging problem faced by wheeled mobile robots (WMRs) such as planetary rovers traversing loose sloped terrain is the inevitable longitudinal slip suffered by the wheels, which often leads to their deviation from the predetermined trajectory, reduced drive efficiency, and possible failures. This study investigates this problem using terramechanics analysis of the wheel-soil interaction. First, a slope-based wheel-soil interaction terramechanics model is built, and an online slip coordinated algorithm is designed based on the goal of optimal drive efficiency. An equation of state is established using the coordinated slip as the desired input and the actual slip as a state variable. To improve the robustness and adaptability of the control system, an adaptive neural network is designed. Analytical results and those of a simulation using Vortex demonstrate the significantly improved mobile performance of the WMR using the proposed control system.

## 1. Introduction

Wheeled mobile robots (WMRs) used in outdoor applications, such as for planetary exploration, often have to navigate loose sloped terrain. WMRs traversing on loose terrain will inevitably encounter the problem of slip between the rigid wheels and the loose soil [1]. An increase in this slip beyond a certain degree will cause the WMR to deviate from the predetermined trajectory and the power consumption to increase and possibly cause failures. Such adverse incidents have been reported for WMRs traversing on loose terrain. For example, the 2005 Opportunity Rover relapsed into loose sand dunes of Purgatory and took five weeks to escape [2]. Similarly, the 2009 Spirit Rover was jammed into the sandy soil of the Home Plate plateau of Mars, owing to which its “mobile” life ended and it became a fixed research platform [3]. The conventional control mechanism of a WMR is designed mainly for navigation in indoor environments, typically by assuming the wheel-terrain interaction as being rigid, that is, without considering the slip or considering it an external disturbance [4]. Indubitably, this assumption of rigidity is reasonable when the velocity and acceleration of WMRs are small or the surface of the traversing path is hard (both of which correspond to a large coefficient of friction) [5]. However, a WMR's wheel-soil mechanics is substantially different in outdoor applications such as planetary exploration [6, 7], and, hence, the conventional rigidity-based control mechanism could cause loss of control of the WMR [8]. Thus, the control of WMRs on loose terrain is faced with unique challenges.

With this background, many researchers have focused on wheel-soil mechanics and considered it to be an important factor for achieving proper control of WMRs traversing on loose terrain and therefore conducted extensive research in this direction. For example, Iagnemma and Dubowsky verified through numerical analysis and tests that the rigidity-based conventional motion planning and control algorithm can cause severe slip and sinkage problems in WMRs [1]; they then modeled wheel-soil mechanics based on terramechanics [9] and also established the multiple-physical-based control mechanism for the navigation of WMRs on flat, loose terrain [9]. Further, Yoshida et al. extended the multiple-physics-based approaches and proposed a traction control method for reducing the slip ratio to a small value [10]. The method was verified through tests on dry sand, thus confirming that the wheel-slip-based control mechanism is effective in preventing severe sinkage and energy waste [10]. Furthermore, in order to avoid incoordination among the wheels, Baumgartner et al. proposed a velocity synchronization algorithm [11]; they verified through experiments using field integrated design and operation rovers that the proposed method can reduce the required power and wheel slippage [11]. Ding et al. proposed an online soil parameter estimation method using the linear least-squares method [12] and a modified, simplified model for wheel-terrain interaction [13], and they compared the simplified model with the original one by numerical analysis [14] to proof the precision of the modified, simplified model. Now, with the development of wheel-soil interaction terramechanics for WMRs, the influence of the wheel slip ratio on the energy consumption and traction efficiency of WMRs is well understood [15]. Moreover, it has become feasible to develop more efficient control algorithms for both minimizing the energy consumption and compensating for the traction efficiency loss of WMRs as caused by wheel slip. However, current research is mainly focused on flat, loose terrain [16–19], and research on the control of WMRs traversing loose sloped terrain is rare. The presently available wheel-soil interaction mechanics models are too complex for control design [9]. The wheel-soil interaction model developed previously [10] involves numerous unknown dynamic time-varying parameters, which easily causes computation delay and control error. Therefore, the design of an effective control mechanism based on wheel-soil mechanics is considered a key research issue in the control of WMRs on loose terrain.

To this end, the present study investigates longitudinal slip-ratio-coordinated control of WMRs while they are climbing up a loose slope, through the planning and tracking of the slip based on an analysis of the wheel-soil interaction mechanism. First, experimental motion analysis is conducted to establish a model for the wheel-soil interaction mechanism. Second, an online planning algorithm for slip ratio that is based on the goal of optimizing drive efficiency is built. Third, a dynamic model is developed for a six-wheel mobile robot climbing loose sloped terrain for the design of its control mechanism. Furthermore, a tracking control method is proposed, wherein individual wheels' slip ratios are used as state variables and the planning slip ratios obtained by the drive efficiency optimization algorithm are used as the desired input. To improve the robustness and adaptability of the proposed tracking control method, an adaptive neural network designed with a weight error in its weight rate is used. Finally, the stability of the control law is proved using the Lyapunov method. Full-scale simulations are performed in real time using the simulation platform RoSTDyn for a six-wheel lunar rover climbing loose sloped terrain to demonstrate the effectiveness of the proposed control scheme.

The rest of this paper is organized as follows.  Section 2 presents the development of a slope-based wheel-soil dynamic model and experimental motion analysis is conducted to amend the model for the wheel-soil interaction mechanism.  Section 3 describes the establishment of a slip planning algorithm based on the goal of drive efficiency optimization.  Section 4 presents a simplified model used for the mobility control of a planetary rover.  Section 5 describes the establishment of an equation of state using the optimal slip as the desired input and the real slip as a state variable; a coordinated slip tracking system is designed using an adaptive neural network.  Section 6 demonstrates the effectiveness of the developed control system via full-scale simulations performed using a six-wheel robot climbing sloped deformable terrain. Finally, Section 7 concludes the study.

## 2. Slope-Based Wheel-Soil Dynamic Interaction Model

When WMRs traverse loose sloped terrain, the wheels may undergo rolling, slip, and sliding movements. The slip ratio is usually used to describe wheel slip and sliding and is expressed as follows:

(1) $$\begin{array}{c} s=\frac{r_{s}\omega -v}{v_{l}}\;v_{l}=\{\begin{array}{cc} r_{s}\omega , & \forall r\omega \geq v\\ v, & \forall r\omega <v, \end{array} \end{array}$$

where *s* is the slip ratio, which means the wheel slip ratio in our study; *ω* is the actual angular velocity of the wheel; *r*_*s*_ is the effective radius of the wheel; *v*_*l*_ is the theoretical translational velocity of the wheel axis; and *v* is the actual translational velocity of the wheel axis. From (1), one can see that −1 ≤ *s* ≤ 1. The condition *s* = 0 indicates pure rolling, wherein the wheel touches the ground at the wheel's instantaneous velocity center; the relationship *v* = *rω* is satisfied in this condition. The condition *s* = 1 indicates that the wheels are in a pure slip state, that is, corresponding to time when *v* = 0. When −1 < *s* < 1, the wheel is in the slip state, and, at this time, *v* < *rω*. When −1 ≤ *s* < 0, the wheel is in the sliding state.

**Figure 1:**
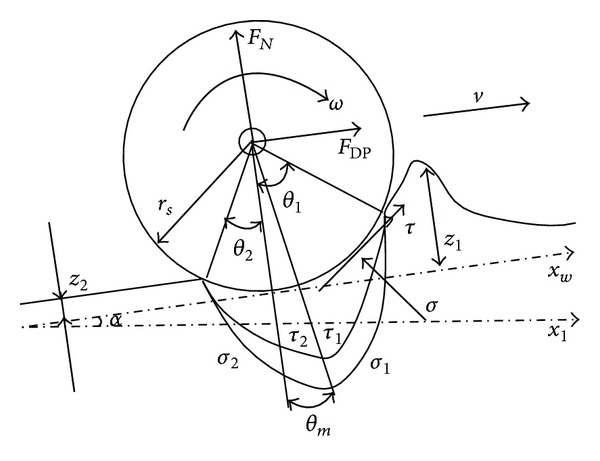
Wheel-soil interaction mechanism on loose sloped terrain.

When the robot is climbing loose sloped terrain, the wheel-soil interaction is as shown in Figure 1. Here, *F*_*N*_ is the normal force, *F*_DP_ is the draw pull force, and *T* is the driving torque. Further, *θ*_1_ denotes the wheel-soil interaction entry angle, *θ*_2_ denotes the departure angle, and *θ*_*m*_ denotes the angle of maximum normal stress. *τ* and σ are the shear stress and normal stress, respectively, when the wheel-soil interaction occurs at any point on the surface; *z* is the wheel sinkage, and *α* is the slope angle.

On the basis of the Reece formula [15], the normal stress is expressed as

(2) $$\begin{array}{c} \sigma =\left(k_{1}+k_{2}b\right){\left(\frac{h}{b}\right)}^{n}, \end{array}$$

where *k*_1_, *k*_2_ are soil bearing characteristic parameters, *h* is the sinkage, *n* is the soil deformation index, and *b* is the wheel width.

Considering the influence of slope angle on the distribution of stress on the surface, the normal stress model for a robot climbing a loose slope is expressed as follows:

(3) σ1(θ)=(k1b+k2)(rcos⁡α)n′(cos⁡θ−cos⁡θ1)n′(θm≤θ≤θ1),σ2(θ)=(k1b+k2)(rcos⁡α)n′ ×{cos⁡[θ1−θ−θ2θm−θ2(θ1−θm)]−cos⁡θ1}n′(θ2≤θ≤θm).

*θ*_*m*_ is calculated by the empirical formula in (4). *n*′ is the modified index for the condition of a robot climbing loose sloped terrain. These variables are calculated as

(4) $$\begin{array}{c} \theta_{m}=\left(c_{1}+c_{2}s\right)\theta_{1}, \end{array}$$

(5) $$\begin{array}{c} \theta_{2}=c_{3}\theta_{1}, \end{array}$$

(6) $$\begin{array}{c} n^{\prime }=c_{1}+c_{2}+c_{3}. \end{array}$$

In (6), *c*_1_, *c*_2_ are constants determined by soil properties. Generally, *c*_1_ ≈ 0.35, 0 ≤ *c*_2_ ≤ 0.25. *c*_3_ is the correction coefficient and is usually in the range 0 ≤ *c*_3_ ≤ 0.075 [15].

On the basis of the Janosi formula [15], we can determine the tangential stress, when the wheel is climbing the loose sloped terrain, as follows:

(7) $$\begin{array}{c} \tau \left(\theta \right)=\left[c+\sigma \left(\theta \right)\tan\varphi \right]\left\{1-\exp\left[-\frac{j\left(\theta \right)}{K}\right]\right\},\\ j\left(\theta \right)=r\left[\left(\theta_{1}-\theta \right)-\left(1-s\right)\left(\sin\theta_{1}-\sin\theta \right)\right]. \end{array}$$

In (7), *c* denotes the soil cohesion coefficient, *φ* denotes the soil friction angle, and *K* denotes the soil shear modulus of deformation.

Through the analysis of the wheel-soil interaction on loose sloped terrain, the correction equation for the concentrated force or torque of the wheel for WMRs climbing such a terrain can be established as

(8) $$\begin{array}{c} F_{N}=rb\overset{\theta_{1}}{\underset{\theta_{2}}{\int }}\sigma \left(\theta \right)\cos\theta \;\text{d}\theta +rb\overset{\theta_{1}}{\underset{\theta_{2}}{\int }}\tau \left(\theta \right)\sin\theta \;\text{d}\theta =G\cos\alpha . \end{array}$$

In the horizontal direction, the balance equation is expressed as follows:

(9) $$\begin{array}{c} F_{\text{DP}}=F_{H}-F_{R}=m\dot{v}. \end{array}$$

Here, *F*_*H*_ is the soil thrust force and *F*_*R*_ is the resistance force, and these are expressed, respectively, as

(10) $$\begin{array}{c} F_{H}=rb\overset{\theta_{1}}{\underset{\theta_{2}}{\int }}\tau \left(\theta \right)\cos\theta \;\text{d}\theta , \end{array}$$

(11) $$\begin{array}{c} F_{R}=rb\overset{\theta_{1}}{\underset{\theta_{2}}{\int }}\sigma \left(\theta \right)\sin\theta \;\text{d}\theta +G\sin\alpha . \end{array}$$

Then, the drawbar pull force can be obtained by the summation of *F*_*H*_ and *F*_*R*_ as

(12) $$\begin{array}{c} F_{\text{DP}}=-rb\overset{\theta_{1}}{\underset{\theta_{2}}{\int }}\sigma \left(\theta \right)\sin\theta \;\text{d}\theta -G\sin\varphi +rb\overset{\theta_{1}}{\underset{\theta_{2}}{\int }}\tau \left(\theta \right)\cos\theta \;\text{d}\theta . \end{array}$$

According to the balance of Euler equations for each wheel, we get

(13) $$\begin{array}{c} T_{i}-T_{Ri}=I_{Wi}{\dot{\omega }}_{Wi}. \end{array}$$

Here, *T*_*i*_ is the motor driving torque and *T*_*Ri*_ is the resistance moment, expressed as

(14) $$\begin{array}{c} T_{Ri}=r^{2}b\overset{\theta_{1}}{\underset{\theta_{2}}{\int }}\tau \left(\theta \right)\text{d}\theta . \end{array}$$

## 3. Slip Planning for Six-Wheel Robot Climbing Loose Sloped Terrain

### 3.1. Relationship between Wheel Slip and Key Performance Indexes of WMR

The key performance indexes of a WMR include its traction efficiency (see Figure 18), thrust coefficient and traction coefficient, and drive efficiency. These indexes are indicators of the mobile performance of a WMR. From the literature [20], a linear combination of draw-pull force and the wheel supporting force approximately gives the equivalent driving torque as

(15) $$\begin{array}{c} T=F_{\text{DP}}\cdot l+F_{N}\cdot e\approx F_{\text{DP}}\cdot r_{s}+F_{N}\cdot e, \end{array}$$

(16) $$\begin{array}{c} F_{\text{DP}}\approx \frac{T}{r_{s}}-F_{N}\cdot \frac{e}{r_{s}}. \end{array}$$

Here, *F*_*t*_ = *T*/*r*_*s*_ is the thrust force, which is produced by the soil deformation caused by the rotation of the motor-driven wheel. *F*_*N*_ · *e*/*r*_*s*_ is the resistance of the soil, and *e*/*r*_*s*_ is the wheel resistance coefficient that reflects the resistance of the soil to prevent the rotation of the wheel. The wheel traction is the drawbar pull force minus the soil resistance generated by the traction. The wheel-soil interaction generates draw-pull force that drives the movement of the robot.

Equation (16) can be transformed as

(17) $$\begin{array}{c} \frac{F_{\text{DP}}}{F_{t}}+F_{N}\cdot \frac{e_{s}}{rF_{t}}\approx 1. \end{array}$$

Let PE = *F*_DP_/*F*_*t*_ = *F*_DP_ *r*_*s*_/*T* denote the traction efficiency. It indicates how much driving force transforms into the effective draw-pull force. From (17), it is understood that the force generated by the motor rotation and thrust force is partly used to generate traction force and partly to overcome the resistance of the soil. Dividing (16) by the normal load *F*_*N*_ gives

(18) $$\begin{array}{c} \frac{F_{\text{DP}}}{F_{N}}\approx \frac{T}{\left(F_{N}r_{s}\right)}-\frac{e}{r_{s}}. \end{array}$$

Let PC = *F*_DP_/*F*_*N*_ be the traction coefficient, which indicates the draw-pull force for providing traction under a unit load, and let TC = *T*/(*F*_*N*_ *r*_*s*_) be the thrust coefficient, which indicates the motor-provided thrust force under a unit load. Then, we have

(19) $$\begin{array}{c} \text{PC}\approx \text{TC}-\frac{e}{r_{s}},\\ \text{PE}=\frac{\left(F_{\text{DP}}/F_{N}\right)}{\left(T/F_{N}r_{s}\right)}=\frac{\text{PC}}{\text{TC}}. \end{array}$$

TE is an important index that denotes the wheel's drive efficiency. Combining with (1), we can express TE as

(20) $$\begin{array}{c} \text{TE}=F_{\text{DP}}\cdot \frac{v}{T\omega }=F_{\text{DP}}\cdot \frac{r_{s}\left(1-s\right)}{T}=\text{PE}\left(1-s\right). \end{array}$$

From the above analysis, we can say that the variables PE, PC, TC, and TE reflect the relationship between the WMR's mobile performance and the slip ratio.

To confirm the influence of the slip ratio on the WMR's mobile performance, experiments on the wheel-soil interaction were conducted for the scenario of a WMR climbing a sloped test bed developed at the State Key Laboratory of Robotics and System at Harbin Institute of Technology, China. In the experiment, five kinds of wheels with different dimensions and wheel lugs were used on different loose soils. Tables 1 and 2 list the parameters of the wheels and of the terrain mechanics, respectively, where *r* is the wheel radius, *b* the wheel width, and *h* the lug height. The travelling velocity of the WMR was 10 mm/s.

**Figure 2:**
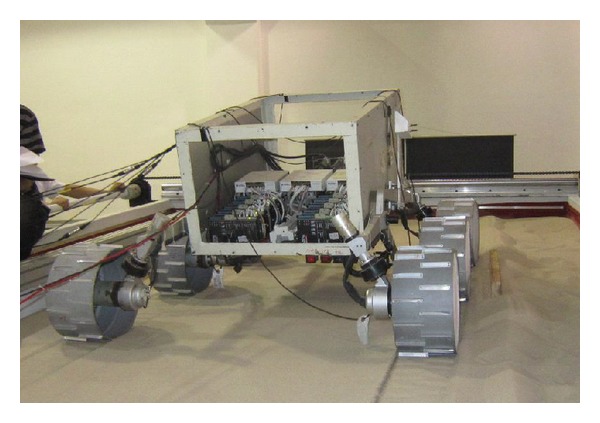
Photograph of six-wheel robot climbing loose sloped terrain in an experiment.

**Figure 3:**
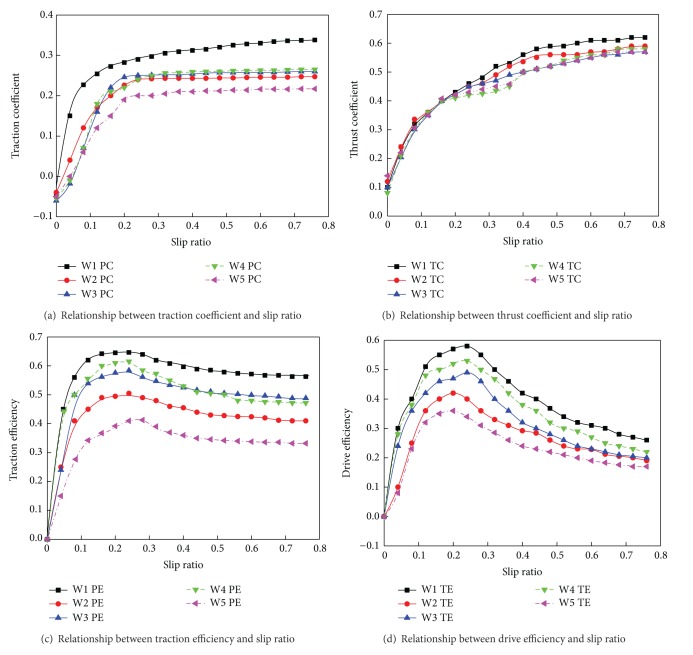
Key performance indexes of robot climbing loose sloped terrain versus wheel slip ratio.

A six-wheel robot was considered an example rover and was made to climb loose sloped terrain angled at 0°, 5°, 10°, 15°, 20°, and 25° to study the relationship between the slip ratio and the key performance indexes (Figure 2).  Figure 3 shows the relationship of the performance indexes PE, PC, TC, and TE with the wheel slip ratio in this climbing scenario, with different ground mechanics parameters and different wheel parameters.

From Figure 3, we can see that the key performance indexes of the WMR are optimal at a wheel slip ratio in the range of 0.1 to 0.4. The index TE is a function of PE, PC, and TC; therefore, if we use an optimization algorithm for TE, we can perform dynamic programming at each wheel slip ratio to ensure the least possible wheel sinkage and thus obtain maximal TE of the WMR.

### 3.2. Slip Ratio Planning Algorithm Based on Optimal Drive Efficiency

When robots climb loose sloped terrain with constant angular control (CAC), each parameter of the wheel-soil mechanics is likely to be different, implying that the wheel slip ratios would also possibly be different. This would inevitably lead to “incoordination” between wheels. Therefore, we aim to optimize the drive efficiency for achieving the desired wheel slip ratio and to control the drive torque of all wheels in a coordinated manner, to be able to track the desired slip ratio and reduce the wheel sinkage. Through tracking of the desired slip ratio for coordinating the energy distributions of each wheel, the drive efficiency of all the wheels can be ensured to be maximal. If the wheel width, radius, payload, and contact angle of the wheel with the terrain are known for a particular WMR, it is possible to determine the desired wheel slip ratio for climbing loose sloped terrain through drive efficiency optimization. The slip ratio planning goal based on drive efficiency optimization is as follows: minimum energy consumption and maximum drive efficiency for a unit running distance of a WMR. The following factors/conditions need to be considered to meet this goal: (1) the force equilibrium equation, (2) maintenance of contact of each of the wheels with the ground and a normal force greater than zero, (3) a soil resistance moment less than the maximum motor torque, and (4) wheel-soil mechanics. Based on the factors/conditions of optimal TE, the expected slip ratio planning algorithm for a target WMR, that is,

(21) $$\begin{array}{c} \min\;J\left(s_{i}\right)=\overset{6}{\underset{i=1}{\sum }}\left|\frac{d\left(F_{\text{DP}i}r_{s}\left(1-s_{i}\right)/T_{i}\right)}{ds_{i}}\right|, \end{array}$$

should meet the following constraints:

(22) s.t. Ti=MRi≤Timax⁡|si|<1FDPi=FDPi(θ1i,θ2i)FNi=FNi(θ1i,θ2i)Ti=Ti(θ1i,θ2i) i=1,…,6.

**Figure 4:**
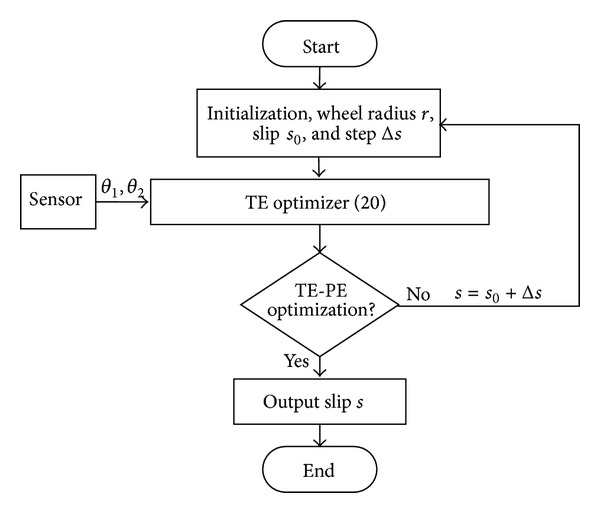
Flowchart of desired slip ratio based on optimal drive efficiency.

When the ground mechanics parameters are known, the flowchart of the planning algorithm for the optimal-TE-based desired slip ratio is as shown in Figure 4.

## 4. Dynamics Modeling of Six-Wheel Robot Climbing Loose Sloped Terrain Based on Wheel-Soil Mechanics

**Figure 5:**
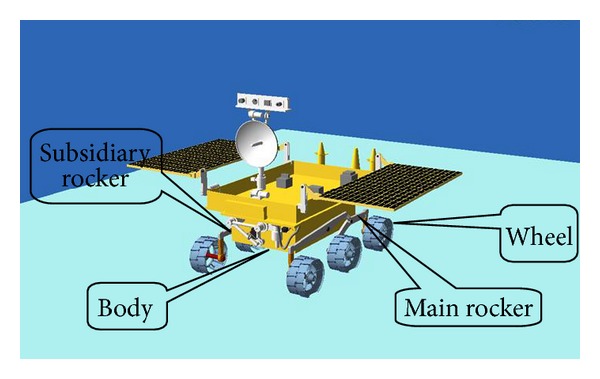
Schematic of six-wheel rocker-type mobile robot.

An optimal-TE-based slip ratio can ensure minimum energy consumption and maximum driving efficiency. To design the tracking control law for the optimal-TE-based desired slip ratio that would be in agreement with the actual slip ratio, we first perform dynamics modeling of climbing of WMRs on loose sloped terrain based on wheel-soil mechanics. Here, again, a six-wheel mobile robot is the research object; owing to its six independent drives and rocker mechanism, it can passively adapt to loose sloped terrain.  Figure 5 shows the schematic of the six-wheel rocker-type mobile robot.

This mobile robot has the following features when climbing loose terrain: (a) when the robot is in longitudinal motion, the system dynamics model is a linear combination of the wheel-soil mechanics; (b) the robot forces on the left and right sides influence each other; (c) sinkage of wheels caused by wheel slip causes the tilting of the robot body.

**Figure 6:**
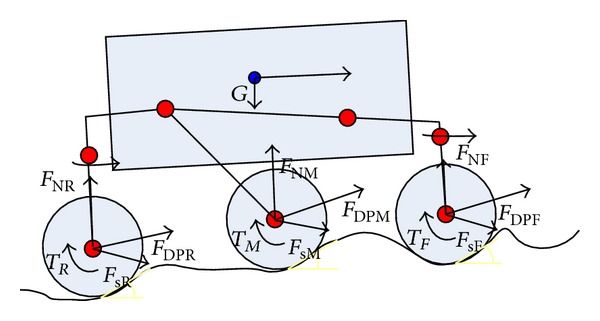
Lateral view of dynamics model based on wheel-soil mechanics during climbing of WMR on loose sloped terrain.

Figure 6 shows the dynamics model based on the wheel-soil mechanics in the case of the six-wheel robot climbing loose sloped terrain.

Equation (23) represents the control-based wheel-soil dynamics model for a six-wheel robot while climbing up deformable slopes with longitudinal slip; it was obtained by the simplification of (9), (12), and (13):

(23) mv˙=∑i=16FDPi(θ1i,θ2i)−Gsin(α+Δα),IWω˙Wi=Ti−TRi(θ1i,θ2i).

Δ*α* is the sinkage incline angle, |Δ*α*| ≤ *ζ*, and *ζ* is a constant. A small *ζ* implies linearized derivation, sin(*α* + Δ*α*) = sin*α* + sinΔ*α*, and, using (11)–(13),

(24) s˙i=−(1−si)[∑i=16FDPi(θ1i,θ2i)−Gsin(α+Δα)]Mv +rs(1−si)2[Ti−TRi(θ1i,θ2i)]IWv.

Next, we define the output function as

(25) h(x)=[x1x2x3x4x5x6]T=[s1s2s3s4s5s6]T.

Combining (23) and (24), we can obtain the standard form of affine nonlinear systems as

(26) $$\begin{array}{c} \dot{x}=f\left(x\right)+g\left(x\right)u+d\left(x\right),\\ h\left(x\right)=x, \end{array}$$

where

(27) x=[s1s2s3s4s5s6]T,u=[T1T2T3T4T5T6]T,f(x)=[f1(x)f2(x)f3(x)f4(x)f5(x)f6(x)]T,fi(x)=−(1−si)[∑i=1n=6FDPi(θ1i,θ2i)−Gsinα]Mv −rs(1−si)2TRi(θ1i,θ2i)IWiv,g(x)=[g1⋯g6]  gi(x)=rs(1−si)2IWiv,d(x)=[d1(x)d2(x)d3(x)d4(x)d5(x)d6(x)]T,di(x)=(1−si)GsinΔαMv.

Then, (26), that is, an equation for a multiple-input, multiple-output (MIMO) system, can be decomposed into (28) for six single-input, single-output (SISO) subsystems. Further, we can design a controller for a SISO subsystem:

(28) $$\begin{array}{c} {\dot{x}}_{i}=f_{i}\left(x\right)+g_{i}\left(x\right)u_{i}+d_{i}\left(x\right),\;i=1,\ldots ,6. \end{array}$$

## 5. Radial Basis Function-Based Adaptive Sliding Tracking Control for Desired Slip Ratio

### 5.1. Approximation Properties of Radial Basis Function Network

A radial basis function (RBF) network is a kind of a three-layer forward neural network. The first layer is the input, made up of source nodes. The input vector map is directly connected to the second hidden layer, and the third layer is the output. The hidden layer is a transformation function unit, called a Gaussian function. Generally, an RBF network is expressed as the excitation function of a hidden unit as follows:

(29) $$\begin{array}{c} \phi_{i}\left(\kappa \right)=\exp\left[-\frac{1}{2b_{i}^{2}}{||\kappa -\xi_{i}||}^{2}\right],\;i=1,2,\ldots ,n. \end{array}$$

Here, *n* is the hidden unit number, *κ* is the network input vector, and *ξ*_*i*_ and *b*_*i*_ are the *i*th basis functions for the center and radius, respectively. The output layer node is the *i*th for nodes in the hidden layer output linear weighted sum:

(30) $$\begin{array}{c} y_{i}=\overset{m}{\underset{j=1}{\sum }}w_{ij}\phi_{j}\left(x\right), \end{array}$$

where *w*_*ij*_ is the weight between *ϕ*_*j*_ and *y*_*i*_.

For any continuous nonlinear function*f*(*x*), one can use the approximate representation of the RBF network as

(31) $$\begin{array}{c} f\left(x\right)=W^{T}\phi \left(x\right)+\varepsilon \left(x\right), \end{array}$$

where **W** is the weight matrix of the RBF network and *ε*(*x*) is the reconstruction error of the neural circuits. The literatures [21, 22] have proved that the RBF network can approximate any continuous nonlinear function of arbitrary precision.

### 5.2. RBF-Based Adaptive Sliding Tracking Control Law

For the system given in (28), if *f*_*i*_ and *g*_*i*_ are precisely known, one can design the general sliding mode control system for tracking the desired slip ratio. When WMRs climb loose sloped terrain, the optimal slip ratio tracking error for the *i*th wheel is *e*_*i*_ = *s*_*i*_ − *s*_*id*_, where *s*_*i*_ is the *i*th actual feedback wheel slip ratio and *s*_*id*_ is the wheel's desired slip ratio.

We express the sliding-mode surface as

(32) $$\begin{array}{c} S_{i}=\rho_{i}e_{i}=\rho_{i}\left(s_{i}-s_{id}\right), \end{array}$$

where *ρ*_*i*_ is a proportional coefficient, generally a normal number, and is used to determine the attenuation speed of the tracking error. The ideal sliding mode can be expressed as

(33) $$\begin{array}{c} {\dot{S}}_{i}=\rho_{i}\left({\dot{s}}_{i}-{\dot{s}}_{id}\right)=0. \end{array}$$

In order to meet the arrival condition of sliding mode variable structure control and in the shortest time to reach the sliding mode surface, to ensure high robustness of the system, the following is the improved index-based control law:

(34) $$\begin{array}{c} {\dot{S}}_{i}=-\varepsilon_{i}\left|S_{i}\right|\mathrm{sgn}\left(S_{i}\right)-k_{i}S_{i}. \end{array}$$

Here, *ε*_*i*_ > 0, *K*_*i*_ > 0, and sgn⁡(*S*_*i*_) is a symbolic function. Upon substituting (28) into (33), we obtain the system control law as

(35) $$\begin{array}{c} u_{i}=\frac{1}{\rho_{i}g_{i}\left(x,v\right)}\left[\rho_{i}f_{i}\left(x,v\right)+\varepsilon_{i}\left|S_{i}\right|\mathrm{sgn}\left(S_{i}\right)+k_{i}S_{i}-\rho_{i}s_{di}\right]. \end{array}$$

At this point, consider the following Lyapunov function candidate:

(36) $$\begin{array}{c} V=\overset{6}{\underset{i=1}{\sum }}V_{i},\;V_{i}=\frac{1}{2}S_{i}^{2}. \end{array}$$

Differentiating it gives

(37) V˙i=SiS˙i=Si(−εi|Si|sgn⁡(Si)−kiSi)=−εiSi|Si|sgn⁡(Si)−kiSi2=−εiSi2−kiSi2<0,

where *V* ≥ 0 and $\dot{V}<0$ are guaranteed to be negative, implying *V* → 0 and also *S* → 0 and $\dot{S}\to 0$ as *t* → *∞*. Therefore, global stability is guaranteed by the Lyapunov theorem.

However, in actual circumstances, *f*_*i*_ and *g*_*i*_ contain many unknown parameters, some of which are dynamic time-varying and therefore cannot be known precisely. Hence, this problem imposes a major limitation on the ideal sliding mode controller given in (35). An RBF neural network possesses the property of an approximate arbitrary nonlinear function, which is not applicable for precision parameters. Therefore, we consider using neural networks with dynamic approximate values ${\tilde{f}}_{i}$ and ${\tilde{g}}_{i}$ of variables *f*_*i*_ and *g*_*i*_ with unknown parameters, based on the RBF neural network function approximation theory.

Let us assume that ${\tilde{f}}_{i}$, ${\tilde{g}}_{i}$ have approximation weights *w*_*fi*_, *w*_*gi*_ and that the *f*_*i*_, *g*_*i*_ approximation errors are *ε*_*fi*_ and *ε*_*gi*_, respectively. That is,

(38) $$\begin{array}{c} {\tilde{f}}_{i}\left(x\right)=\tilde{f}\left(x\;|\;w_{fi}\right)=w_{fi}^{T}\phi \left(x\right)+\varepsilon_{fi}, \end{array}$$

(39) $$\begin{array}{c} {\tilde{g}}_{i}\left(x\right)=\tilde{g}\left(x\;|\;w_{gi}\right)=w_{gi}^{T}\phi \left(x\right)+\varepsilon_{gi}. \end{array}$$

Because the RBF neural network is used to approximate an unknown nonlinear function in the system, an approximation error inevitably exists. The neural network fitting error should be reduced as possible, so we define ${\tilde{w}}_{fi}$ and ${\tilde{w}}_{gi}$ as the optimal weights to estimate *w*_*fi*_ and *w*_*gi*_:

(40) $$\begin{array}{c} {\tilde{w}}_{fi}=\arg\;\underset{w_{fi}\in \Omega_{fi}}{\min}\left[\underset{x\in \Omega_{x}}{\sup}|{\tilde{f}}_{i}\left(x\;|\;w_{fi}\right)-f_{i}\left(x\right)\right], \end{array}$$

(41) $$\begin{array}{c} {\tilde{w}}_{gi}=\arg\;\underset{w_{gi}\in \Omega_{gi}}{\min}\left[\underset{x\in \Omega_{x}}{\sup}|{\tilde{g}}_{i}\left(x\;|\;w_{gi}\right)-g_{i}\left(x\right)\right]. \end{array}$$

In (40) and (41), *Ω*_*x*_ ⊂ *R* ^*n*^, *Ω*_*fi*_ and *Ω*_*gi*_ ⊂ *R* ^*m*^. *Ω*_*x*_, *Ω*_*fi*_, and *Ω*_*gi*_ all are compact sets further and *n* and *m* are the input layer and hidden layer nodes, respectively. As a result, ${\tilde{f}}_{i}(x|{\tilde{w}}_{fi})$ and ${\tilde{g}}_{i}(x|{\tilde{w}}_{gi})$ are the optimal approximations of *f*_*i*_(*x*) and *g*_*i*_(*x*), respectively. Then, we define the weights of the neural network error as

(42) $$\begin{array}{c} \eta_{fi}={\tilde{w}}_{fi}-w_{fi},\;\;\eta_{gi}={\tilde{w}}_{gi}-w_{gi}, \end{array}$$

where ${\dot{\eta }}_{fi}={\dot{\tilde{w}}}_{fi}-{\dot{w}}_{fi}$, ${\dot{\eta }}_{gii}={\dot{\tilde{w}}}_{gi}-{\dot{w}}_{gi}$.

Taking the weights of the neural network, *w*_*fi*_ and *w*_*gi*_, as the adaptive control law, we get

(43) $$\begin{array}{c} {\dot{w}}_{fi}=-\gamma_{fi}S_{i}\phi_{fi},\;\;{\dot{w}}_{gi}=\gamma_{gi}S_{i}\phi_{gi}u_{i}. \end{array}$$

Based on (35), (39), (40), and (43), we get the slip ratio tracking control law using RBF neural network adaptive sliding method, expressed as

(44) $$\begin{array}{c} u_{i}=\frac{1}{\rho_{i}{\tilde{g}}_{i}\left(x\right)}\left[\rho_{i}{\tilde{f}}_{i}\left(x\right)+\varepsilon_{i}\left|S_{i}\right|\mathrm{sgn}\left(S_{i}\right)+k_{i}S_{i}-\rho_{i}s_{di}\right]. \end{array}$$

### 5.3. Stability Analysis

Let us define the minimum approximation error of a neural network as

(45) $$\begin{array}{c} \delta_{i}=\left[{\tilde{f}}_{i}\left(x\;|\;{\tilde{w}}_{fi}\right)-f_{i}\left(x\right)\right]+\left[{\tilde{g}}_{i}\left(x\;|\;{\tilde{w}}_{gi}\right)-g_{i}\left(x\right)\right]u_{i}. \end{array}$$

Then, substituting (44) and (45) into (37) gives

(46) S˙i=ρi(fi(x)+gi(x)ui)−ρis˙di=ρi(fi(x)+g~i(x ∣ wgi)ui+gi(x)ui) −g~i(x ∣ wgi)ui−ρis˙di=fi(x)−f~i(x ∣ wfi)+ρix˙di−kisign⁡Si+gi(x)ui −g~i(x ∣ wgi)ui−ρis˙di=fi(x)−f~i(x ∣ wfi)+[gi(x)ui−g~i(x ∣ wgi)ui] −kisgn⁡Si=f~i(x ∣ w~fi)−f~i(x ∣ wfi) +[g~i(x ∣ w~gi)−g~i(x ∣ wgi)]ui−δi−kisgn⁡Si=(w~fiT−wfiT)ϕfi(x)+(w~giT−wgiT)ϕgi(x)ui −δi−kisgn⁡Si=ηfiTϕfi(x)+ηgiTϕgi(x)ui−δi−kisgn⁡Si.

Consider the following Lyapunov function candidate:

(47) $$\begin{array}{c} V=\overset{6}{\underset{i=1}{\sum }}V_{i},\\ V_{i}=\frac{1}{2}\left({S_{i}}^{2}+\frac{1}{\gamma_{fi}}\eta_{fi}^{T}\eta_{fi}+\frac{1}{\gamma_{gi}}\eta_{gi}^{T}\eta_{gi}\right). \end{array}$$

Differentiating it gives

(48) V˙i=SiS˙i+1γfiηfiTη˙fi+1γgiηgiTη˙gi=Si(ηfiTϕfi(x)+ηgiiTϕgii(x)ui−δi−kisgn⁡(Si)) +1γfiηfiTη˙fi+1γgiηgiTη˙gi=SiηfiTϕfi(x)+1γfiηfiTη˙fi+SiηgiTϕgi(x)ui +1γgiηgiTη˙gi−δiSi−Sikisgn⁡(Si)=1γfiηfiT(η˙fi+γfiSiϕfi(x)) +1γgiηgiT(η˙gi+γgiSiϕgi(x)ui)−δiSi−ki|Si|2=−δiSi−ki|Si|2.

Based on the approximation theory of the RBF neural network, as long as numerous hidden layer nodes exist, the adaptive RBF neural network can give an infinitesimal value of the approximation error. Then,

(49) $$\begin{array}{c} {\dot{V}}_{i}\leq 0,\;\;\dot{V}\leq 0. \end{array}$$

Then, to reduce chattering that occurs usually, boundary layer methods are used, wherein a saturation function is employed instead of a signum function:

(50) $$\begin{array}{c} \mathrm{sat}\left(S_{i},\phi_{i}\right)=\{\begin{array}{cc} 1, & S_{i}>\phi_{i}\\ k_{i}S_{i}, & \left|S_{i}\right|\leq \phi_{i},k_{i}\phi_{i}=1\\ -1, & S_{i}<-\phi_{i}, \end{array} \end{array}$$

where *ϕ*_*i*_ is the normal to the boundary layer thickness.

**Figure 7:**
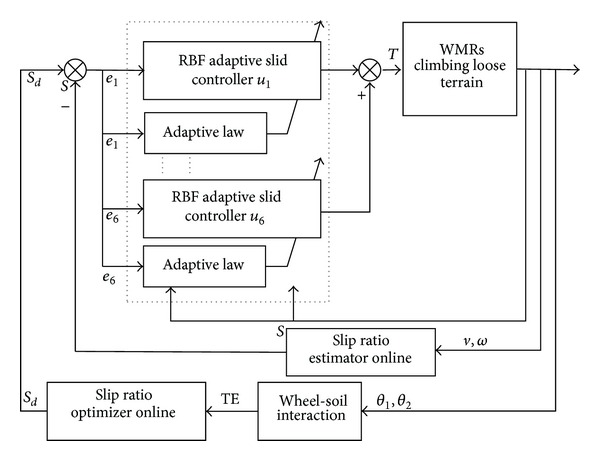
Flowchart for optimal slip ratio tracking control based on RBF neural network adaptive sliding mode.

The flowchart for optimal slip ratio tracking control based on the RBF neural network adaptive sliding mode in the case of a WMR climbing loose sloped terrain is shown in Figure 7.

## 6. Simulation Experiment Based on RoSTDyn Platform

### 6.1. RoSTDyn

**Figure 8:**
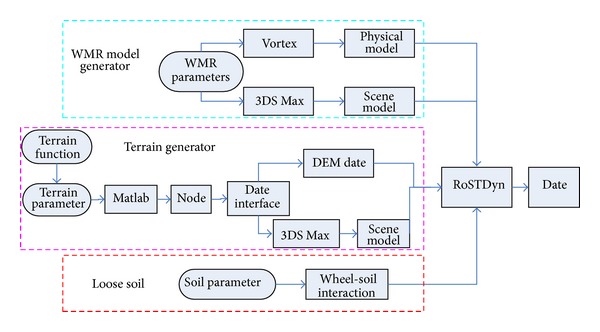
Schematic of simulation using RoSTDyn system.

RoSTDyn is a multibody dynamics simulation platform for WMRs moving on loose sloped terrain; it uses a Vortex engine and VC++ and was developed by the RCAMC Laboratory. Here, VC++ was used to generate the WMR system, terrain module, and wheel-soil interaction mechanics model, and the kinetic function and scene function provided by the Vortex engine were used for calculations and 3D realization. The basic simulation outline is as shown in Figure 8. From the literature [23], it is known that the RoSTDyn robot provides precise simulations on loose sloped terrain through comprehensive testing.

### 6.2. Simulation of Control of Six-Wheel Lunar Rover Climbing Loose Sloped Terrain

Consider a six-wheel lunar rover as an example. When it is climbing a slope on the moon, the most influential factor is the slip ratio of the wheels. Using the proposed control algorithm (RBFAS) to adjust the drive torque to track the desired slip ratio, the lunar rover can effectively climb up a slope with the least possible wheel sinkage and maximum drive efficiency of all the wheels.

This simulation is performed as follows. The lunar rover is started from its initial stationary state. Then, the desired slip ratio based on the optimal TE is input into the slip ratio tracking algorithm. The wheel drive moment is adjusted to control the wheel slip ratio and obtain the optimal slip ratio.

The system parameters for the simulation are set as follows.

- Robot parameters are the following: *M* = 120 kg, *m*_*w*_ = 1.75 kg*·*m^2^, *r* = 0.15 m, *b* = 0.15 m, and *h* = 0.01 m.
- Sandy soil characteristic parameters are the following: *k*_1_ = 1800, *k*_2_ = 820000, *g* = 1.6333 m/s^2^, *c* = 520, *φ* = 42°, *K* = 0.01732, *c*_1_ = 0.35, *c*_2_ = 0.042, and *c*_3_ = 0.012.
- Control parameters are the following: *ρ*_*i*_ = 10, *σ*_*i*_ = 0.17, $b_{i}={\left[\begin{array}{ccccc} 10 & 10 & 10 & 10 & 10 \end{array}\right]}^{T}$, *γ*_*fi*_ = 5, *γ*_*gii*_ = 1, and $w_{fi}=w_{gi}=\left[\begin{array}{cccc} 6 & 6 & 6 & 6 \end{array}\right]$.

Figure 9 shows images of the six-wheel lunar rover climbing the sloped terrain in the RoSTDyn 3D simulation platform. Figures 10, 11, 12, 13, 14, 15, and 16 show the key performance indices of WMR comparisons climbing up a 25° slope under RBFAS and CAC control strategies.

**Figure 10:**
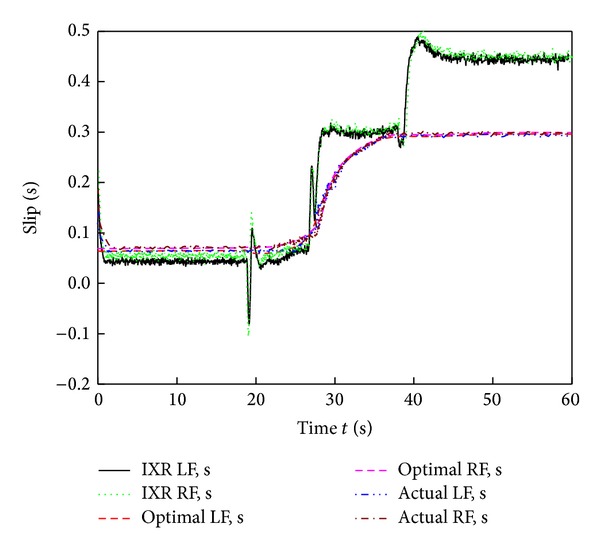
Front-wheels slip ratio comparison of different control strategy.

**Figure 11:**
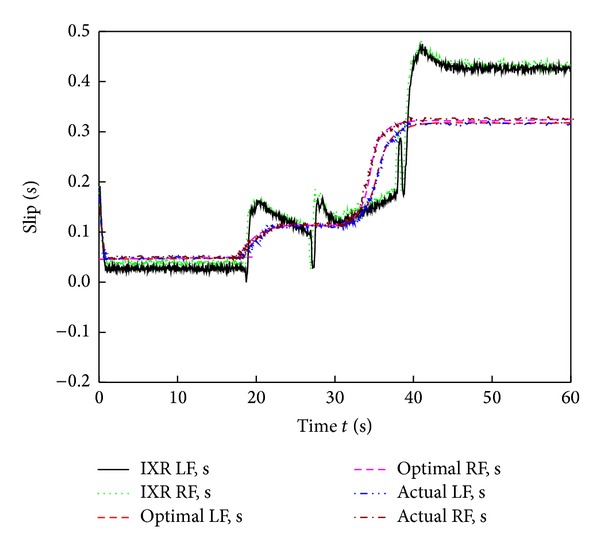
Middle-wheels slip ratio comparison of different control strategy.

**Figure 12:**
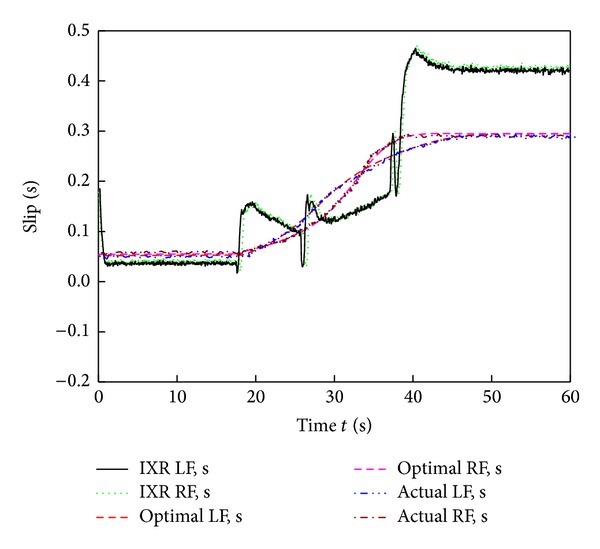
Behind-wheels slip ratio comparison of different control strategy.

**Figure 13:**
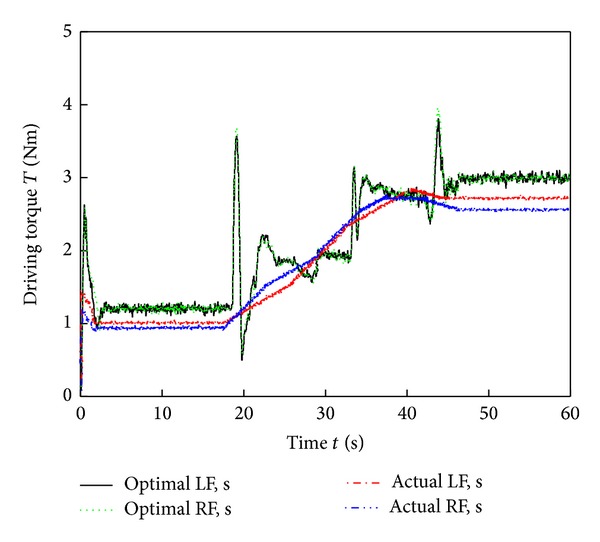
Front-wheels driving torque comparison of different control strategy.

**Figure 14:**
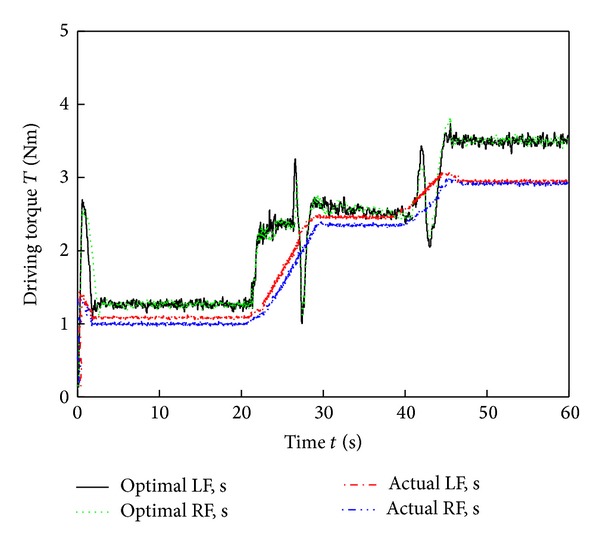
Middle-wheels driving torque comparison of different control strategy.

**Figure 15:**
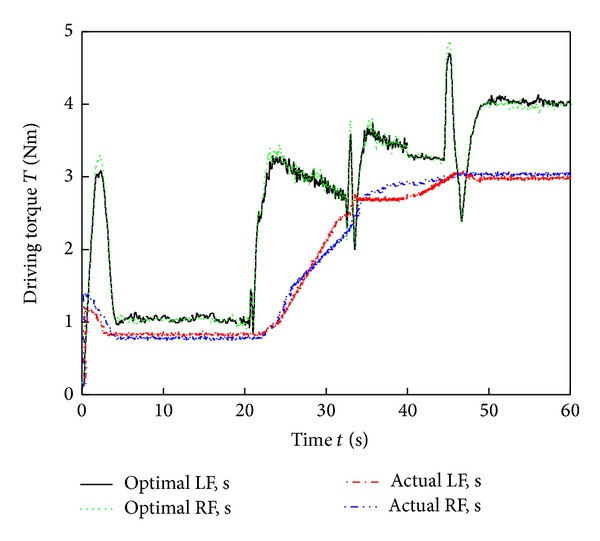
Behind-wheels driving torque comparison of different control strategy.

**Figure 17:**
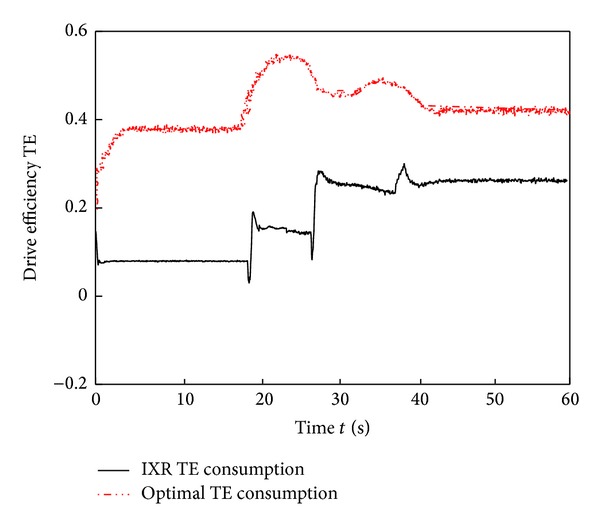
Drive efficiency comparison of different control strategy.

**Figure 18:**
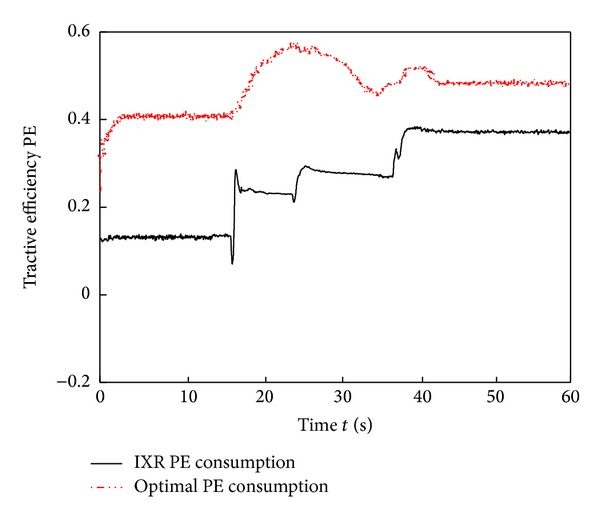
Tractive efficiency comparison of different control strategy.

The simulation results in Figures 10–15 show that the RBFAS control algorithm given in (45) is superior to those using CAC.  Figure 17 shows that the RBFAS control algorithm in (45) significantly reduces the energy consumption and drive efficiency of the six-wheel lunar rover.

## 7. Conclusion

In this study, we analyzed wheel-soil interaction based on traditional terrain mechanics for WMRs climbing loose sloped terrain and determined the influence of key performance indexes of the WMRs on the wheel slip ratio. We developed an online slip ratio planning algorithm based on the optimal drive efficiency (TE) of the WMRs. Next, using the optimal-TE-based slip ratio as the input, the actual slip ratio as the state variable, and the wheel drive moment as the control input, we established a tracking system for the optimal-TE-based slip ratio using the method of nonlinear decoupling design. This was done with the aim of improving the robustness and adaptability of the tracking system. An adaptive neural network was used and a weight error in the weight rate was introduced in this network. The control stability of the system was confirmed using the Lyapunov method. Finally, full-scale simulations were performed to verify that the proposed control scheme not only retains the stability of the system but also improves the robot's mobile performance significantly.

## Figures and Tables

**Figure 9:**
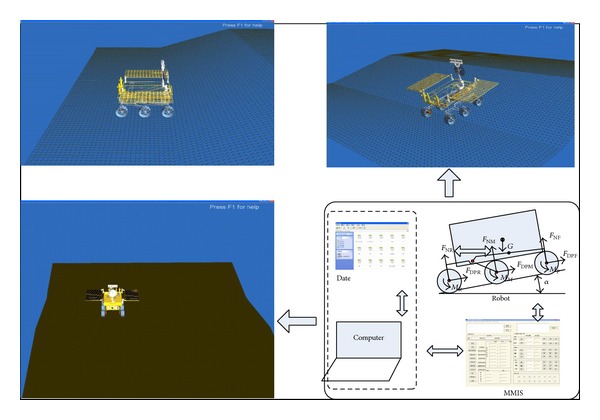
Six-wheel lunar rover climbing sloped terrain in RoSTDyn 3D simulation.

**Figure 16:**
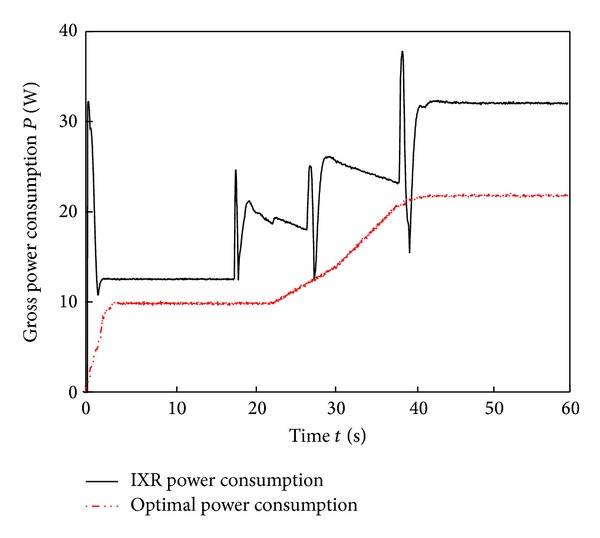
Power consumption comparison of different control strategy.

**Table 1:** Geometrical parameters of wheels.

Wheel	Radius *r* (mm)	Width *b* (mm)	Lug height *h* (mm)
Wh1	157.4	165	15
Wh2	135	110	15
Wh3	135	165	15
Wh4	135	165	10
Wh5	135	165	10

**Table 2:** Parameters of terrain mechanics of sand and lunar soil [14].

Terrain parameter	Sand	Lunar soil
*n*	1.10	1.0
*k*_*c*_ (kPa/m^*n*−1^)	0.95	1.4
*k*_*φ*_ (kPa/m^*n*^)	1528.43	820
*c* (kPa)	1.04	0.52 (0.1~2.7)
*φ* (°)	28	42 (25~50)
*K* (m)	0.0254	0.0178
